# Supplementary material for: Causal interplay between lactose intolerance and gut microbiota: a combined bidirectional Mendelian randomization and in vivo validation study
Source: Front Nutr. 2026 Jun 1;13:1803337. doi: 10.3389/fnut.2026.1803337 (PMC13265576; doi:10.3389/fnut.2026.1803337)
Supplement: Supplementary file 1 [file Data_Sheet_1.zip › supplementary materials/Forward/leave one out cross validation/ebi-a-GCST90027573.finngen_R12_E4_LACTONAS.pdf]

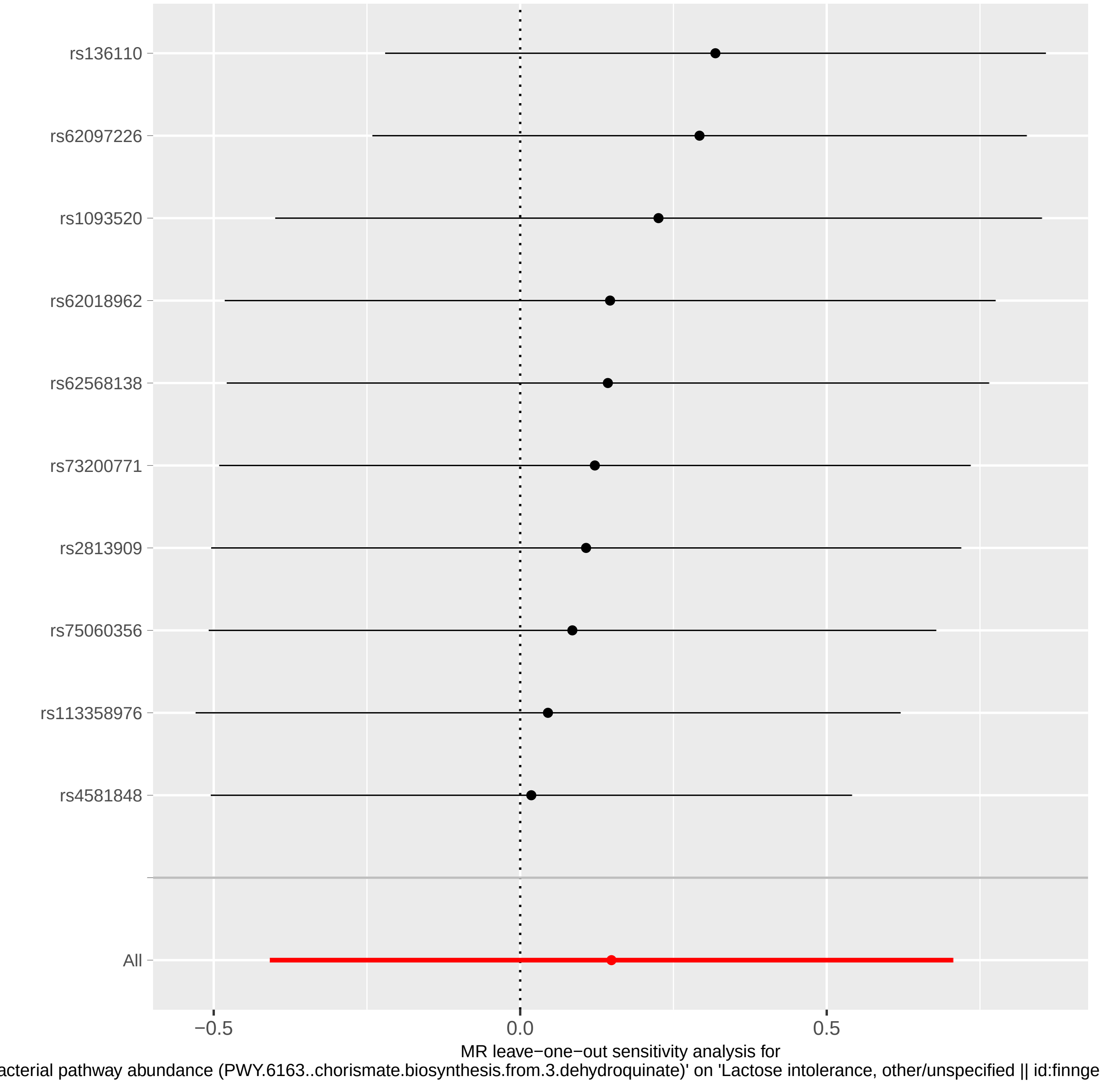

rs136110
rs62097226
rs1093520
rs62018962
rs62568138
rs73200771
rs2813909
rs75060356
rs113358976
rs4581848
All
−0.5
0.0
0.5
MR leave−one−out sensitivity analysis for
acterial pathway abundance (PWY.6163..chorismate.biosynthesis.from.3.dehydroquinate)' on 'Lactose intolerance, other/unspecified || id:finnge
